# Supplementary material for: Could Horizontal Gene Transfer Explain 5S rDNA Similarities Between Frogs and Worm Parasites?
Source: Biomolecules. 2025 Jul 12;15(7):1001. doi: 10.3390/biom15071001 (PMC12294053; doi:10.3390/biom15071001)
Supplement: Supplementary file 1 [file biomolecules-15-01001-s001.zip › biomolecules-3569312-supplementary.pdf]

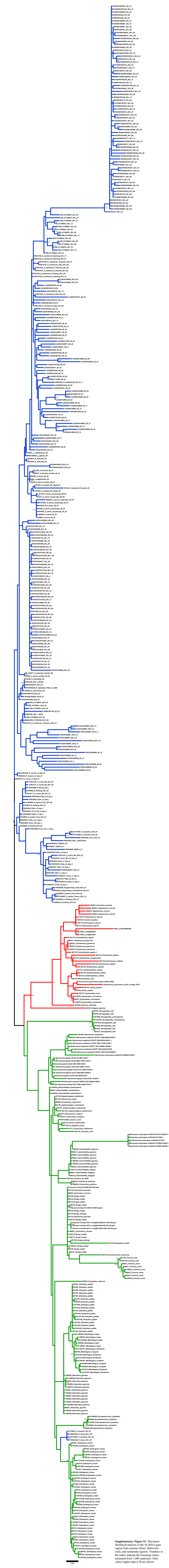

Supplementary Figure S1. Maximum likelihood analysis of the 5S rRNA gene region from anurans (blue), flatworms (red), and nematodes (green). Numbers in the nodes indicate the bootstrap values estimated from 1,000 replicates. Only values higher than 0.50 are shown.

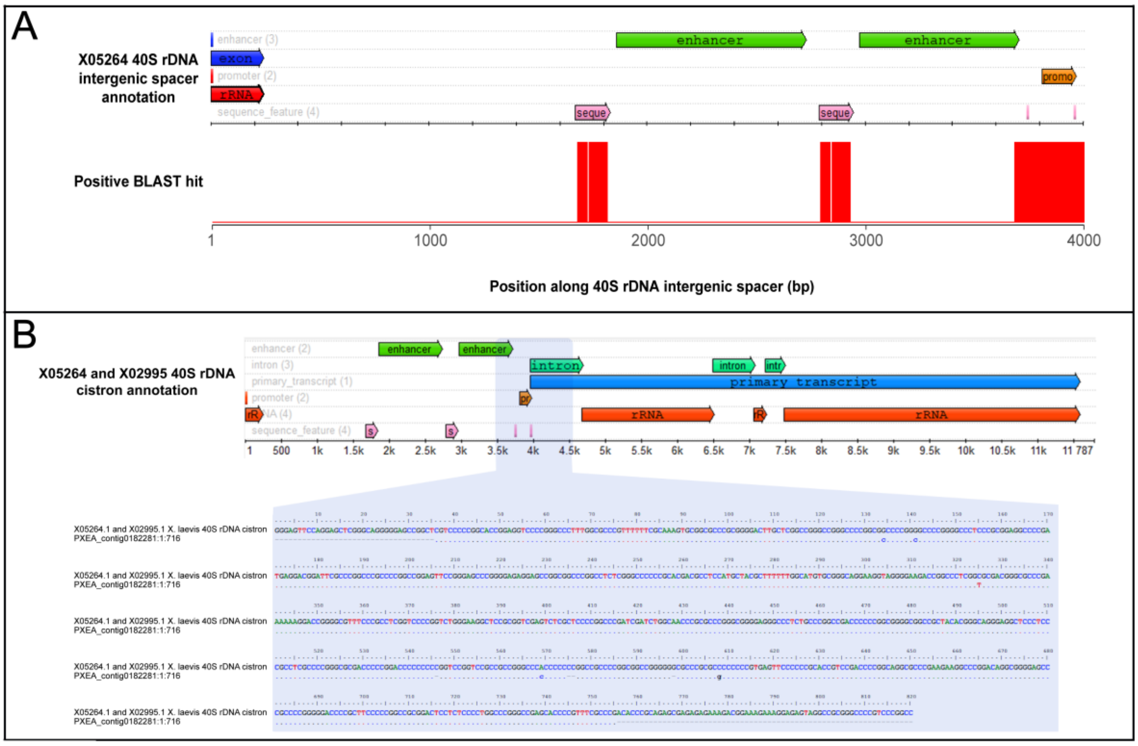

Supplementary Figure S2. A. BLAST search positive hits result for the intergenic spacer of 40S rDNA from *Xenopus laevis* against the *Protopolystoma xenopodis* genome assembly. **B.** Alignment of 40S rDNA of *X. laevis* with the *Pr. xenopodis* contig0182281, which were similar in the BLAST search.

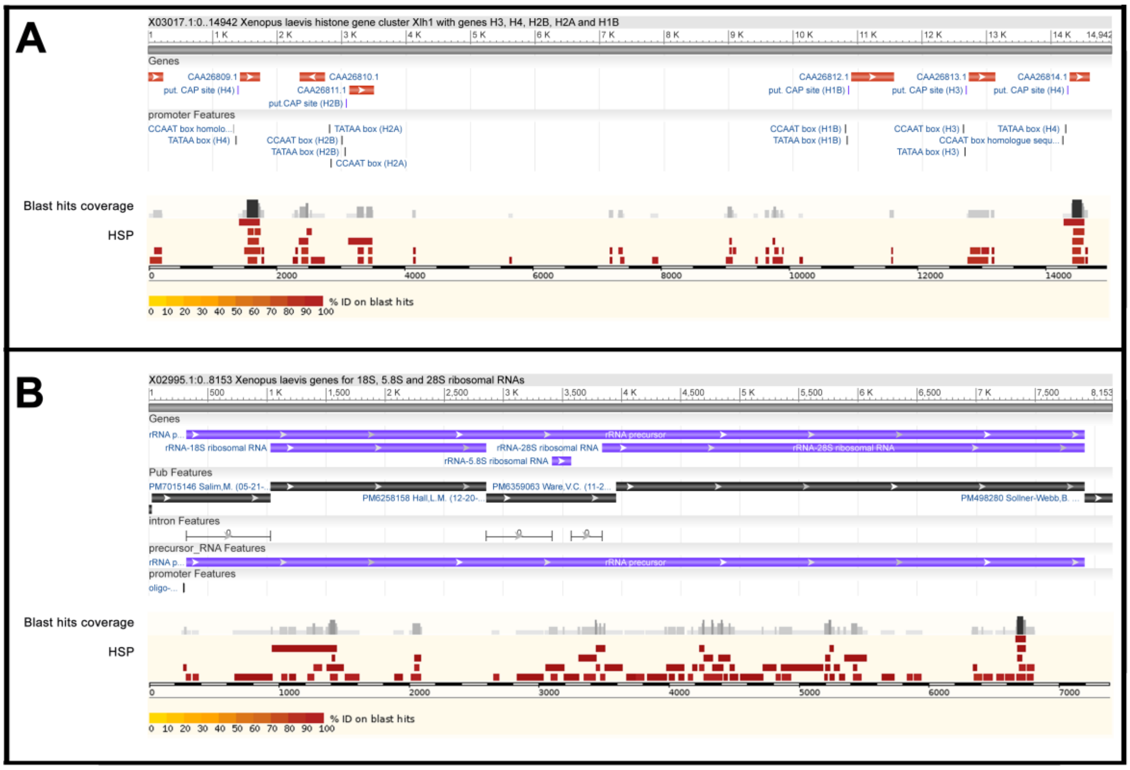

Supplementary Figure S3. Summary of BLAST alignments of the *Xenopus laevis* histone gene cluster (**A**) and the 18S-5.8S-28S rRNA gene cluster (**B**) against the genome assembly of *Protopolystoma xenopodis.* The top graphs indicate the annotation map of each sequence, whereas the bottom graphs show the coverage of the BLAST hits from the *Pr. xenopodis* genome and the percentage identity of the alignments generated from BLAST positive hits.

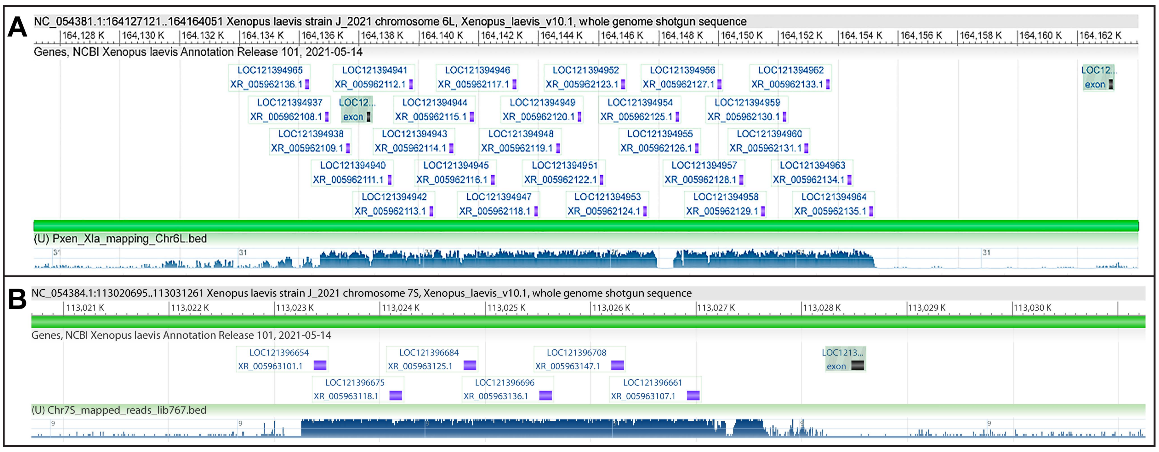

Supplementary Figure S4. Genomic features of the regions in chromosome 6L (**A**) and 7S (**B**) of *Xenopus laevis* that were densely mapped by reads of the *Protopolystoma xenopodis* libraries and show 5S rDNA clusters. The annotated genes are all 5S rRNA genes (LOC121394937, LOC121394938, LOC121394940 - LOC121394949, LOC121394951 - LOC121394960, and LOC121394962 - LOC121394965 in A; LOC121396654, LOC121396675, LOC121396684, LOC121396696, LOC121396708, and LOC121396661 in B). This image was generated in NCBI GenomeViewer.

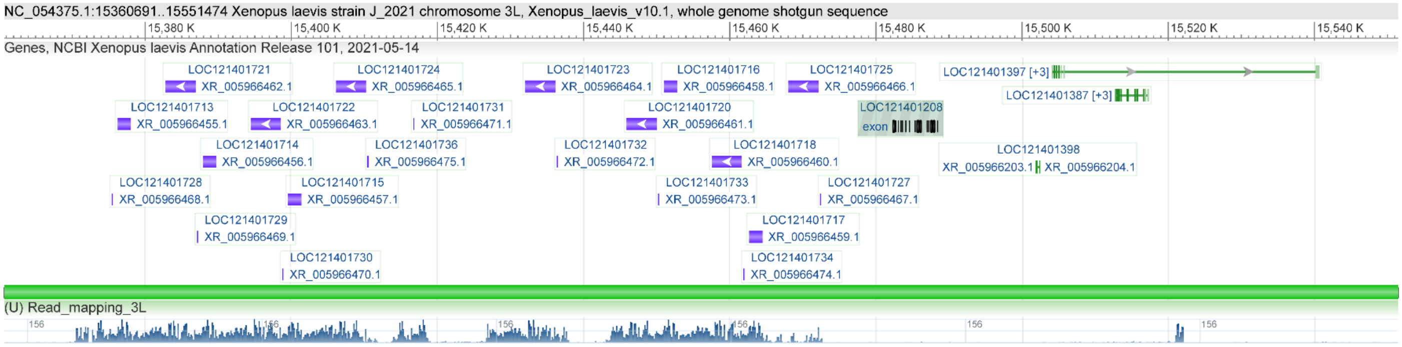

Supplementary Figure S5. Genomic features of the second region in chromosome 3L of *Xenopus laevis* that was densely mapped by reads of the *Protopolystoma xenopodis* libraries. The following genes are annotated: 18S rRNA gene (LOC121401713, LOC121401714, LOC121401715, LOC121401716, and LOC121401717), 5.8S rRNA gene (LOC121401727, LOC121401728, LOC121401729, LOC121401730, LOC121401731, LOC121401732, LOC121401733, LOC121401734, and LOC121401736), and 28S rRNA gene (LOC121401718, LOC121401720, LOC121401721, LOC121401722, LOC121401723, LOC121401724, and LOC121401725). This image was generated in NCBI GenomeViewer.

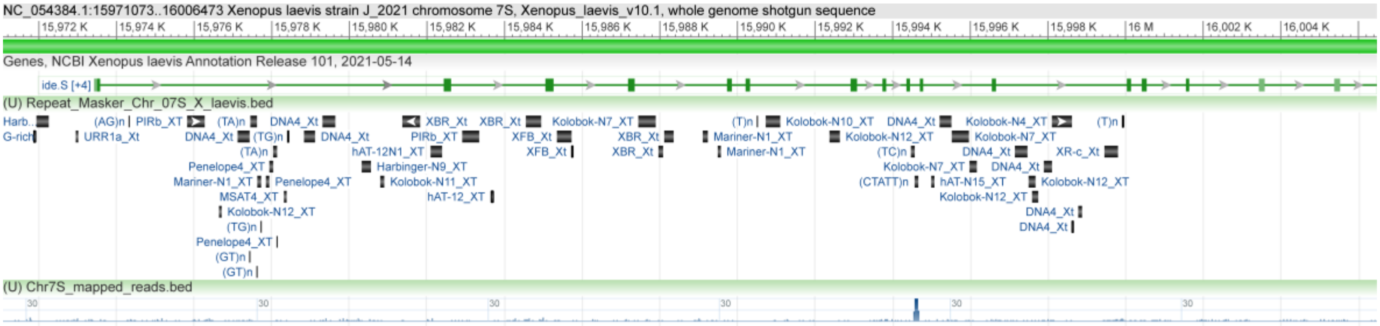

Supplementary Figure S6. Genomic features of the second region in chromosome 7S of *Xenopus laevis* that was densely mapped by reads of the *Protopolystoma xenopodis* libraries. The annotated gene is *ide.S* (XB-GENE-6486175), and a region with a peak of mapped reads coincide with a microsatellite site [(CTATT)n] in an intronic region of this gene. This image was generated in NCBI GenomeViewer.

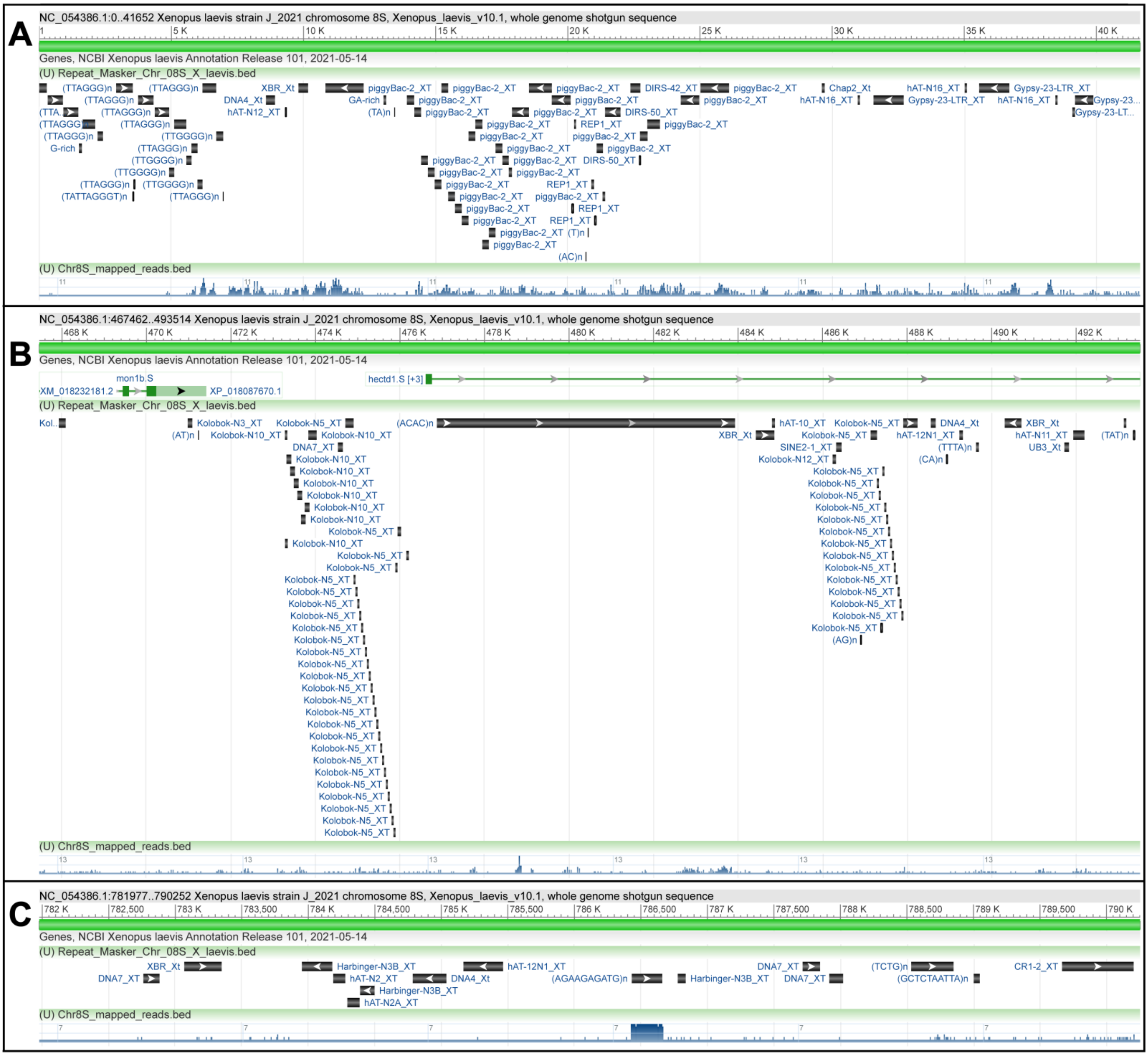

Supplementary Figure S7. Genomic features of the region in chromosome 8S of *Xenopus laevis* that was densely mapped by reads of the *Protopolystoma xenopodis* libraries. Note in the segment from position 1 to position 41,652 (**A**) several regions annotated for piggyBac and telomeric motifs $(TTAGGG)_n$, while between the positions 467,462 and 493,514 (**B**) there is a microsatellite segment $[(ACAC)_{n]}$, which is located in the first intron of the gene *hectd1.S* (XB-GENE-17344034), and between the positons 781,977 and 790,252 (**C**) the mapped reads coincide with the microsatellite motif $(AGAAGAGATG)_n$. These images were generated in NCBI GenomeViewer.

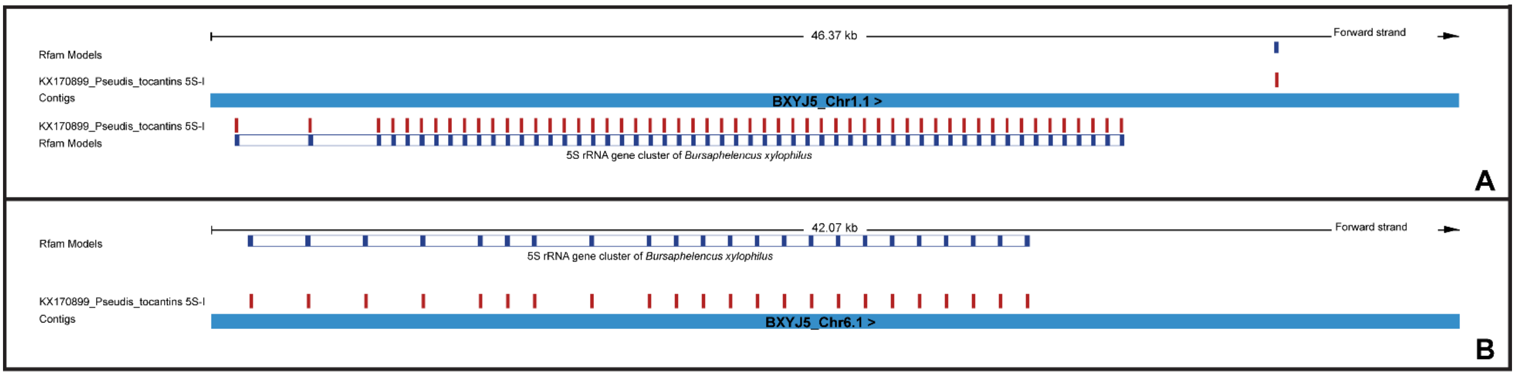

Supplementary Figure S8. Output of the BLAST searches for *Pseudis tocantins* type I 5S rDNA in the genome assembly of *Bursaphelenchus xylophilus* (accession number: GCA_904067135.1). The regions detected by BLAST in chromosomes 1 (**A**) and 6 (**B**) are indicated in red. The blue blocks identify regions annotated as 5S rRNA genes based on Rfam models. This figure was generated using the GenomeBrowser tool provided by the BLAST tool search in WormBase Parasite.

Supplementary Table S1. Sequences of 5S rDNA used in the present study downloaded from GenBank, WormBase Parasite, and 5S rRNA database.

Species	Sequence type	Download source	Accession number
Anura			
Amolops mantzorum	5S rDNA	GenBank	KX913750, KX913753, KX913756 - KX913758, KX913762 and KX913764 - KX913778
Anaxyrus americanus	5S rRNA gene	GenBank	X58365
Cycloramphus bolitoglossus	5S-I rDNA	GenBank	MT920608
	5S-II rDNA	GenBank	MT920607 and MT920615
Dendropsophus soaresi	5S-I rDNA	GenBank	OK634330 and OK634342
	5S-II rDNA	GenBank	OK634342
Engystomops coloradorum	5S-II rDNA	GenBank	OK634298
Engystomops freibergi	5S-I rDNA	Genbank	JF325868 – JF325870
	5S-II rDNA	Genbank	JF325844 and JF325845
Engystomops guayaco	5S-II rDNA	GenBank	OK634299
Engystomops petersi	5S-I rDNA	Genbank	JF325859, JF325866 and JF325867
	5S-II rDNA	Genbank	JF325847 and JF325858
Gastrotheca riobambae	5S rDNA	Genbank	M74438
Leptobrachium leishanense	5S-I rDNA	Genome assembly	RXON01001063, RXON01001346, RXON01001513, RXON01001911, RXON01002687, RXON01002717, RXON01002855, RXON01003919 and RXON01004421
	5S-II rDNA	Genome assembly	RXON01003679
	5S-III rDNA	Genome assembly	RXON01003679
Lithobates catesbeianus	5S rRNA gene	Genbank	X58367
	5S-I rDNA	Genome assembly	LIAG020140602, LIAG020573420, LIAG020679224, LIAG020741645, LIAG021057541, LIAG021107393, LIAG021248571 and LIAG021715894
	5S-II rDNA	Genome assembly	LIAG020140602
Lithobates pipiens	5S rRNA gene	Genbank	X58368
Lysapsus limellum	5S-II rDNA	GenBank	OK634325
Nanorana parkeri	5S rRNA gene	Genbank	NW_017306541, NW_017306611, NW_017308120, NW_017308844 and NW_017328351
Oophaga pumilio	5S-I rDNA	Genome assembly	VIAB01001185
Pelophylax lessonae	5S rRNA gene	Genbank	FJ572051
Pelophylax ridibundus	5S rRNA gene	Genbank	FJ572052
Pleurodema diplolister	5S-I rDNA	GenBank	OK634296
	5S-II rDNA	GenBank	OK634318 and OK634319
	5S-III rDNA	GenBank	OK634320
Physalaemus albifrons	5S-I rDNA	GenBank	OK634282
	5S-II rDNA	GenBank	OK634307 and OK634309
Physalaemus albonotatus	5S-I rDNA	GenBank	OK634285 and OK634287
	5S-II rDNA	GenBank	OK634306
Physalaemus centralis	5S-I rDNA	GenBank	OK634288
	5S-II rDNA	GenBank	OK634304
Physalaemus cicada	5S-II rDNA	GenBank	OK634311
Physalaemus cuvieri	5S-I rDNA	Genbank	JF281127, JF281128, JF281130, JF281131, OK634291 and OK634301
	5S-II rDNA	Genbank	JF281132 and OK634301
	5S-IV rDNA	GenBank	OK634321 and OK634322
Physalaemus ephippifer	5S-I rDNA	GenBank	OK634293
	5S-II rDNA	GenBank	OK634310
Physalaemus kroyeri	5S-IV rDNA	GenBank	OK634323
Physalaemus nattereri	5S-I rDNA	GenBank	OK634295
	5S-II rDNA	GenBank	OK634317
Pseudis bolbodactyla	5S-I rDNA	GenBank	OK634324
Pseudis fusca	5S-I rDNA	GenBank	OK634326 and OK634329
Pseudis tocantins	5S-I rDNA	Genbank	KX170899 and KX170901
	5S-II rDNA	Genbank	KX170905 and KX170906
Pyxicephalus adspersus	5S rDNA	Genom assembly	PZQJ01000006 and PZQJ01005048
Rana temporaria	5S-I rDNA	Genome assembly	VIAC01044233, VIAC01055848 and VIAC01061615
	5S-II rDNA	Genome assembly	VIAC01040632 and VIAC01046807
Rhinella marina	5S-I rDNA	Genome assembly	ONZH01002077
	5S-II rDNA	Genome assembly	ONZH01002077
Spea multiplicata	5S-I rDNA	Genome assembly	VKOC01002425, VKOC01001145, VKOC01001608, VKOC01002194, VKOC01002415, VKOC01002496, VKOC01003025, VKOC01004017, VKOC01007104, VKOC01014844, VKOC01014901, VKOC01015599, VKOC01017253, VKOC01017838, VKOC01018097, VKOC01039654, VKOC01047229, VKOC01047817 and VKOC01048368
	5S-II rDNA	Genome assembly	VKOC01015292

Xenopus borealis	5S rDNA	Genbank	K01374, K01537, V01425 and V01426
Xenopus laevis	5S rDNA	Genbank	J01009, J01010, J01012, J01898, J01899, M10027, M10635 and M63899
	5S rRNA gene	Genbank	K02695, M10676, M10850, M30904, M35055, M35175 and M35176
Xenopus tropicalis	5S rDNA	Genbank	X12622, X12623, X12624, NR_033270 and NR_033271
Plathyhelminthes			
Clonorchis sinensis	5S rRNA gene	5S rRNA database	E00487
Dugesia japonica	5S rRNA gene	5S rRNA database	E00167
Echinococcus granulosus	5S rRNA gene	WormBase Parasite	EGRAN001_EgG_scaffold_0020 and ASM52419v1_EG_S00103
Echinostoma caproni	5S rRNA gene	5S rRNA database	E01713 – E01715
Fasciola hepatica	5S rRNA gene	5S rRNA database	E01410
Hymenolepis microstoma	5S rRNA gene	5S rRNA database	E00757 – E00759
Hymenolepis nana	5S rRNA gene	5S rRNA database	E01727
Mesocestoides corti	5S rRNA gene	5S rRNA database	E01729
Opisthorchis viverrini	5S rRNA gene	5S rRNA database	E02672 – E02674
Planocera reticulate	5S rRNA gene	Genbank	X01550
Protopolystoma xenopodis	5S rRNA gene	WormBase Parasite	CAAALY010007052, CAAALY010013821, CAAALY010082537 and CAAALY010184163
Schistocephalus solidus	5S rRNA gene	5S rRNA database	E01737 – E01739
Schistosoma japonicum	5S rRNA gene	5S rRNA database	E00371 – E00374
Schistosoma margrebowiei	5S rRNA gene	5S rRNA database	E01731
Schistosoma mattheei	5S rRNA gene	5S rRNA database	E01732
Schistosoma rodhaini	5S rRNA gene	5S rRNA database	E01733 – E01736
Taenia asiatica	5S rRNA gene	5S rRNA database	E01740 and E01741
Trichobilharzia regenti	5S rRNA gene	5S rRNA database	E01718 – E01719
Nematoda			
Ancylostoma ceylanicum	5S rRNA gene	5S rRNA database	E00690
Angiostrongylus cantonensis	5S rRNA gene	5S rRNA database	E01720 and E01721
Angiostrongylus costaricensis	5S rRNA gene	5S rRNA database	E01722
Ascaris lumbricoides	5S rRNA gene	5S rRNA database	E01723
Ascaris suum	5S rRNA gene	5S rRNA database	E00032
Bursaphelenchus xylophilus	5S rRNA gene	5S rRNA database	E00466 – E00470
Brugia malayi	5S rRNA gene	5S rRNA database	E02116 – E02128
Brugia pahangi	5S rRNA gene	5S rRNA database	E01724 and E01725
Caenorhabditis brenneri	5S rRNA gene	5S rRNA database	E00323
Caenorhabditis briggsae	5S rRNA gene	5S rRNA database	E00031 and E00316
Caenorhabditis elegans	5S rRNA gene	5S rRNA database	E00129, E02114 and E02115
Caenorhabditis japonica	5S rRNA gene	5S rRNA database	E00324 – E00330
Caenorhabditis tropicalis	5S rRNA gene	5S rRNA database	E00401
Dracunculus medinensis	5S rRNA gene	5S rRNA database	E01711
Dictyocaulus viviparous	5S rRNA gene	5S rRNA database	E01373 and E01374
Elaeophora elaphi	5S rRNA gene	5S rRNA database	E01726
Globodera pallida	5S rRNA gene	5S rRNA database	E01189 – E01205
Haemonchus placei	5S rRNA gene	5S rRNA database	E01728
Heterodera glycines	5S rRNA gene	5S rRNA database	E00357 – E00370
Heterorhabditis bacteriophora	5S rRNA gene	5S rRNA database	E00437 – E00439
Meloidogyne floridensis	5S rRNA gene	5S rRNA database	E01268 – E01273
Meloidogyne hapla	5S rRNA gene	5S rRNA database	E00383 – E00387
Meloidogyne incognita	5S rRNA gene	5S rRNA database	E00388 – E00392
Nippostrongylus brasiliensis	5S rRNA gene	5S rRNA database	E01730
Onchocerca flexuosa	5S rRNA gene	WormBase Parasite	UZAJ01012249
	5S rRNA gene	WormBase Parasite	UZAJ01000351 and UZAJ01000212
Onchocerca volvulus	5S rRNA gene	5S rRNA database	E00810 and E00811
Oscheius sp.	5S rRNA gene	5S rRNA database	E01708
Panagrellus redivivus	5S rRNA gene	5S rRNA database	E00623
Pristionchus pacificus	5S rRNA gene	5S rRNA database	E00393 and E00394
Romanomermis culicivorax	5S rRNA gene	5S rRNA database	E01973
Steinernema scapterisci	5S rRNA gene	5S rRNA database	E01296
	5S rRNA gene	WormBase Parasite	KN167482, KN167747, KN167554 and KN166783
Strongyloides ratti	5S rRNA gene	5S rRNA database	E01975 – E01980
Strongyloides venezuelensis	5S rRNA gene	5S rRNA database	E01969 and E01970
Subanguina moxae	5S rRNA gene	5S rRNA database	E01809 – E01848
Syphacia muris	5S rRNA gene	5S rRNA database	E01710
Toxocara canis	5S rRNA gene	5S rRNA database	E01742
Trichuris muris	5S rRNA gene	5S rRNA database	E00913 – E00918
Trichuris suis	5S rRNA gene	5S rRNA database	E01149 and E01150
Wuchereria bancrofti	5S rRNA gene	5S rRNA database	E01743 and E01744

Supplementary Table S2. Blast searches using the contig0184163 from the *Protopolystoma xenopodis* genome assembly as query against genomes of selected species representing distinct classes of flatworms. Only the highest score alignments are shown for each species.

Flatworms (BioProject)	***P. xenopodis* contig0184163**			
	Length of the alignment (bp)	*Alignment similarity (%)*	*E-value*	*Annotation*
Clonorchis sinensis (PRJDA72781)	79	84.8	1.3e-6	5S rRNA
Dibothriocephalus latus (PRJEB1206)	48	91.7	3.4e-7	5S rRNA
Echinococcus granulosus (PRJEB121)	44	90.9	8.2e-5	5S rRNA
Echinostoma caproni (PRJEB1207)	79	86.1	5.5e-9	5S rRNA
Fasciola hepatica (PRJEB25283)	79	86.1	5.5e-9	5S rRNA
Fasciola hepatica (PRJNA179522)	74	86.5	2.2e-8	5S rRNA
Gyrodactylus salaris (PRJNA244375)	80	98.8	7.7e-36	5S rRNA
Macrostomum lignano (PRJNA284736)	38	97.4	2.2e-8	5S rRNA
Mesocestoides corti (PRJEB510)	28	89.6	8.2e-5	5S rRNA
Schistocephalus solidus (PRJEB527)	48	91.7	3.4e-7	5S rRNA
Schistosoma mansoni (PRJEA36577)	53	88.7	2.1e-5	5S rRNA
Spirometra erinaceieuropaei (PRJEB1202)	48	89.6	8.2e-5	5S rRNA
Trichobilharzia regenti (PRJEB4662)	48	91.7	3.4e-7	5S rRNA

Supplementary Table S3. Sequences from *Xenopus laevis* used to investigate possible contaminants in the *Protopolystoma xenopodis* genome assembly.

**Gene or sequence name**	**Genbank/DFAM accession number**
RAG-1	EF535914, EF535887, NM_001172083, L19324,
H3A	X72950, X72949, X03104, BG354652, BG023354, J00983, X03017
U1	NM_001088134, X12430, BC108447
18S-5.8S-28S rDNA	X02995
Ribosomal gene spacer	X05264
Actin-A type 8	M24770
RHOD	L07770, U23808
Harbinger	DR000240199
Tc1-Mariner	DR000240244
DIRS	DR000240200
Gypsy	DR000240201
CR1	DR000240205

Supplementary Table S4. BLAST searches using repetitive multigene families and single copy sequences from *Xenopus laevis* as queries against the *Protopolystoma xenopodis* genome assembly.

X. laevis gene (accession number)	*P. xenopodis* contig:position	Alignment similarity (%)	Alignment length (bp)	E-value
RAG1.S mRNA (NM_001172083)	none	-	-	-
RAG1 (EF535914)	none	-	-	-
RAG1 (EF535887)	none	-	-	-
RAG1 (L19324)	none	-	-	-
RAG1 (NM_001349446)	none	-	-	-
RAG1 (AY874341)	none	-	-	-
RAG1 (AY874315)	none	-	-	-
RAG1 (KF724646)	none	-	-	-
RAG1 (KF724644)	none	-	-	-
Histone core genes (X03017)				
Histone H3l (X72950)	PXEA_contig0018273:3145-3359	81.4	215	9.4e-22
Histone H3r (X72949)	PXEA_scaffold0016045:2910-3035	83.3	126	9.4e-15
Histone H3 (X03104)	PXEA_contig0018273:3157-3473	80.8	317	1.4e-32
Histone H3 (J00983)	PXEA_contig0018273:3157-3248	87.0	92	1.3e-15
Histone H3Al (NM_001098432)	PXEA_scaffold0016045:2734-2933	79.5	200	1.5e-10
Histone H3 (J00984)	PXEA_scaffold0016045:2910-3035	84.0	126	3.0e-19
U1 snoRNA (NM_001088134)	none	-	-	-
U1 snoRNA (X12430)	none	-	-	-
U1 snoRNA (BC108447)	none	-	-	-
40S rDNA intergenic spacer (X05264)	PXEA_contig0182281:399-716	99.1	318	4.8e-171
40S rDNA intergenic spacer (X05264)	PXEA_contig0182281:452-534	94.0	83	4.9e-26
40S rDNA intergenic spacer (X05264)	PXEA_contig0182281:543-586	93.2	44	1.5e-07
40S rDNA gene region (X02995)	PXEA_contig00167724: 292-786	100	495	0
40S rDNA gene region (X02995)	PXEA_contig00167724: 1-290	99.7	290	2.8e-161
40S rDNA gene region (X02995)	PXEA_contig00231293: 135-461	100	327	0
Actin type 8 (M24770)	PXEA_contig0015028:1161-1333	83.8	173	4.4e-25
Rhodopsin (L07770)	none	-	-	-
Rhodopsin (U23808)	PXEA_contig0192600:124-410	89.9	287	3.4e-90
p53 (X77546)	none	-	-	-

Supplementary Table S5. BLAST searches for representative transposable elements and 18S-5.8S-28S rDNA from *Xenopus laevis* against the *Protopolystoma xenopodis* genome assembly.

TE class (query size (bp)/rDNA	*Pr. xenopodis* contig:location (bp)	Query cover (%)	Alignment size (bp)	Alignment score	E-value	Similarity (%)
Harbinger (487)	CAAALY010088168:686-779	19.09	94	54	1.2e-21	89.4
	CAAALY010088168:782-779	11.08	55	51	7.5e-20	98.2
	CAAALY010088168:442-503	12.52	62	50	3e-19	95.2
	CAAALY010088168:655-689	6.98	35	27	1.6e-05	94.3
	CAAALY010088168:825-855	6.16	31	24	0.0009	93.35
	CAAALY010088168:458-495	7.59	38	22	0.015	89.5
	CAAALY010197217:114-161	9.65	48	28	4e-06	89.6
CR1 (4617)	CAAALY010042203:319-364	0.97	46	38	4.2e-11	95.7
	CAAALY010042203:364-393	0.63	30	26	0.0006	96.7
	CAAALY010042203:410-497	1.88	88	60	3.1e-24	92
	CAAALY010059076:1821-1926	2.27	106	90	3.9e-42	96.2
	CAAALY010081295:1018-1122	2.25	105	45	2.8e-15	85.7
	CAAALY010081295:1134-1230	2.08	97	61	7.9e-25	90.7
	CAAALY010081295:1233-1277	0.95	45	33	4e-08	93.3
	CAAALY010081295:1307-1392	1.84	86	50	2.9e-18	89.5
	CAAALY010120749:118-209	1.97	92	36	6.6e-10	84.8
	CAAALY010120749:330-450	2.60	121	41	6.8e-13	83.5
	CAAALY010120749:466-508	0.91	43	35	2.6e-09	95.3
	CAAALY010151870:1-724	15.66	724	670	0	98.1
	CAAALY010151870:769-846	1.67	78	74	1.4e-32	98.7
	CAAALY010151870:845-874	0.63	30	26	0.0006	96.7
	CAAALY010196424:187-214	0.58	28	24	0.0095	96.4
	CAAALY010019370:276-308	0.69	33	29	9.9e-06	97.0
	CAAALY010019370:344-414	1.52	71	35	2.6e-09	87.3
	CAAALY010019370:949-989	0.87	41	25	0.0024	90.2
	CAAALY010039777:1-116	3.57	166	146	1.5e-75	97
	CAAALY010039777:167-219	1.13	53	53	4.7e-20	100
	CAAALY010039777:219-301	1.78	83	63	5.1e-26	94
	CAAALY010070212:1-202	4.35	202	182	4.9e-97	97.5
	CAAALY010070212:590-655	1.41	66	58	4.9e-23	97
DIRS (4948)	CAAALY010033820:168-194	0.53	27	23	0.04	96.3

	CAAALY010033820:256-292	0.73	37	25	0.0026	91.9
	CAAALY010048564:2281-2309	0.57	29	29	1.1e-05	100
Gypsy (824)	CAAALY010210624:106-189	10.07	84	68	9.2e-30	95.2
	CAAALY010210624:522-605	10.07	84	73	9.6e-33	96.4
	CAAALY010043528:716-795	9.59	80	49	2e-18	90
	CAAALY010043528:978-1018	4.85	41	33	7.1e-09	95.1
	CAAALY010043528:1019-1082	7.65	64	37	2.9e-11	89.1
	CAAALY010043528:1162-1228	8.01	67	43	7.6e-15	91
	CAAALY010043528:1547-1646	12.01	100	64	2.2e-27	91
rDNA (8153)	CAAALY00001305:828-849	0.27	22	22	0.24	100
	CAAALY00092869:732-778	0.58	47	35	4.20E-09	93.6
	CAAALY00092869:823-868	0.56	46	26	0.00097	89.1
	CAAALY00100351:1128-1327	2.45	200	188	2.10E-100	98.5
	CAAALY00100351:1-43	0.53	43	43	7.00E-14	100
	CAAALY00100351:161-311	1.85	151	135	8.80E-69	97.4
	CAAALY00100351:28-162	1.66	135	135	8.80E-69	100
	CAAALY00100351:336-396	0.75	61	41	1.10E-12	91.8
	CAAALY00100351:411-474	0.78	64	52	3.00E-19	95.3
	CAAALY00100351:522-652	1.61	131	115	7.60E-57	96.9
	CAAALY00100351:654-724	0.87	71	71	1.40E-30	100
	CAAALY00100351:724-855	1.62	132	128	1.30E-64	99.2
	CAAALY00100351:854-967	1.40	114	102	4.30E-49	97.4
	CAAALY00100351:972-1111	1.72	140	140	9.10E-72	100
	CAAALY00167724:1-290	3.56	290	290	2.80E-161	100
	CAAALY00167724:292-786	6.07	495	495	0	100
	CAAALY00188043:426-457	0.39	32	28	6.20E-05	96.9
	CAAALY00208758:123-158	0.44	36	36	1.10E-09	100
	CAAALY00208758:158-227	0.86	70	70	5.40E-30	100
	CAAALY00208758:227-302	0.93	76	72	3.50E-31	98.7
	CAAALY00208758:304-357	0.66	54	54	1.90E-20	100
	CAAALY00208758:32-65	0.42	34	26	0.00097	94.1
	CAAALY00208758:358-421	0.78	64	64	2.10E-26	100
	CAAALY00208758:441-611	2.10	171	171	2.90E-90	100
	CAAALY00208758:67-99	0.40	33	33	6.50E-08	100
	CAAALY00228254:14-103	1.10	90	90	6.30E-42	100
	CAAALY00228254:224-389	2.04	166	166	2.80E-87	100

CAAALY00228254:390-432	0.53	43	43	7.00E-14	100
CAAALY00228254:432-469	0.47	38	38	6.70E-11	100
CAAALY00228254:470-502	0.40	33	33	6.50E-08	100
CAAALY00228254:503-540	0.47	38	38	6.70E-11	100
CAAALY00231293:135-461	4.01	327	323	0	99.7
CAAALY00231293:1-96	1.18	96	92	4.00E-43	99
CAAALY00231293:476-513	0.47	38	38	6.70E-11	100
CAAALY00231293:514-537	0.29	24	24	0.015	100
CAAALY00231293:97-129	0.40	33	25	0.0039	93.9
CAAALY00000006:25211-25250	0.49	40	28	6.20E-05	92.5
CAAALY00000614:7303-7362	0.74	60	60	5.00E-24	100
CAAALY00001485:6453-6506	0.66	54	42	2.80E-13	94.4
CAAALY00001485:6526-6599	0.91	74	42	2.80E-13	89.2
CAAALY00007369:2158-2219	0.76	62	22	0.24	83.9
CAAALY00007369:2227-2299	0.90	73	41	1.10E-12	89
CAAALY00007369:2675-2727	0.65	53	41	1.10E-12	94.3
CAAALY00007369:2738-2807	0.86	70	46	1.10E-15	91.4
CAAALY00007369:2829-2889	0.75	61	45	4.50E-15	93.4
CAAALY00011849:4496-4522	0.33	27	23	0.06	96.3
CAAALY00014915:3530-3556	0.33	27	23	0.06	96.3
CAAALY00018746:1947-2006	0.74	60	36	1.10E-09	90
CAAALY00022960:115-169	0.67	55	35	4.20E-09	90.9
CAAALY00022960:1-70	0.86	70	70	5.40E-30	100
CAAALY00026525:1531-1570	0.49	40	36	1.10E-09	97.5
CAAALY00026525:1-70	0.86	70	50	4.60E-18	92.9
CAAALY00026525:597-626	0.37	30	30	4.00E-06	100
CAAALY00026525:644-672	0.36	29	29	1.60E-05	100
CAAALY00026525:695-765	0.87	71	51	1.20E-18	93
CAAALY00026525:775-832	0.71	58	38	6.70E-11	91.4
CAAALY00026525:835-867	0.40	33	25	0.0039	93.9
CAAALY00028316:2562-2596	0.43	35	23	0.06	91.4
CAAALY00033018:2-44	0.53	43	27	0.00025	90.7
CAAALY00033018:64-94	0.38	31	23	0.06	93.5
CAAALY00033018:96-118	0.28	23	23	0.06	100
CAAALY00037688:1045-1089	0.55	45	25	0.0039	88.9
CAAALY00037688:478-506	0.36	29	21	0.94	93.1

	CAAALY00037688:514-544	0.38	31	23	0.06	93.5
	CAAALY00037688:548-638	1.12	91	47	2.90E-16	87.9
	CAAALY00046634:865-904	0.49	40	28	6.20E-05	92.5
	CAAALY00046634:89-109	0.26	21	21	0.94	100
	CAAALY00046634:916-969	0.66	54	34	1.60E-08	90.7
	CAAALY00049746:502-522	0.26	21	21	0.94	100
	CAAALY00052662:1062-1105	0.54	44	36	1.10E-09	95.5
	CAAALY00052662:1130-1166	0.45	37	21	0.94	89.2
	CAAALY00052662:571-678	1.32	108	68	8.40E-29	90.7
	CAAALY00052662:687-812	1.55	126	102	4.30E-49	95.2
	CAAALY00052662:70-129	0.74	60	52	3.00E-19	96.7
	CAAALY00052662:860-916	0.70	57	37	2.70E-10	91.2
	CAAALY00052662:917-961	0.55	45	21	0.94	86.7
	CAAALY00070485:1-48	0.59	48	48	7.30E-17	100
Histone gene cluster (14942)	CAAALY00004465:4116-4141	0.17	26	22	0.48	96.2
	CAAALY00009885:1419-1452	0.23	34	22	0.48	91.2
	CAAALY00010396:3724-3746	0.15	23	23	0.12	100
	CAAALY00018273:3157-3473	2.12	317	69	4.30E-29	80.4
	CAAALY00018273:3160-3334	1.17	175	51	2.40E-18	82.3
	CAAALY00026773:2177-2207	0.21	31	27	0.0005	96.8
	CAAALY00026773:2332-2385	0.36	54	22	0.48	85.2
	CAAALY00035130:2033-2126	0.63	94	26	0.002	81.9
	CAAALY00086015:240-262	0.15	23	23	0.12	100
	CAAALY00103135:708-738	0.21	31	23	0.12	93.5
	CAAALY00103931:550-580	0.21	31	23	0.12	93.5
	CAAALY00126429:553-611	0.39	59	23	0.12	84.7
	CAAALY00126429:697-773	0.52	77	37	5.40E-10	87
	CAAALY00138576:647-953	2.05	307	163	3.50E-85	88.3
	CAAALY00138576:649-953	2.04	305	153	3.20E-79	87.5
	CAAALY00154349:730-857	0.86	128	28	0.00013	80.5
	CAAALY00154349:730-857	0.86	128	32	5.20E-07	81.2
	CAAALY00154781:162-192	0.21	31	23	0.12	93.5
	CAAALY00154781:68-102	0.23	35	31	2.10E-06	97.1
	CAAALY00189299:655-677	0.15	23	23	0.12	100
	CAAALY00197894:3-369	2.46	367	199	1.10E-106	88.6
	CAAALY00202208:286-362	0.52	77	33	1.30E-07	85.7

CAAALY00202208:448-506	0.39	59	23	0.12	84.7
CAAALY00225288:147-277	0.88	131	63	1.60E-25	87
CAAALY00225288:278-334	0.38	57	29	3.20E-05	87.7
CAAALY00225288:342-543	1.35	202	86	3.10E-39	85.6
CAAALY00225288:84-109	0.17	26	26	0.002	100
CAAALY00225288:84-109	0.17	26	26	0.002	100
CAAALY00225288:84-112	0.19	29	29	3.20E-05	100
CAAALY00225288:87-109	0.15	23	23	0.12	100
CAAALY00225288:87-110	0.16	24	24	0.031	100
CAAALY00242198:449-474	0.17	26	26	0.002	100
CAAALY00242198:449-474	0.17	26	26	0.002	100
CAAALY00242198:449-477	0.19	29	29	3.20E-05	100
CAAALY00242198:452-475	0.16	24	24	0.031	100
CAAALY00242198:452-479	0.19	28	24	0.031	96.4
CAAALY00001423:2999-3069	0.48	71	23	0.12	83.1
CAAALY00001423:2999-3177	1.20	179	31	2.10E-06	79.3
CAAALY00001423:3104-3177	0.50	74	26	0.002	83.8
CAAALY00002566:8529-8770	1.62	242	58	1.60E-22	81
CAAALY00002566:8607-8770	1.10	164	56	2.50E-21	83.5
CAAALY00006079:4940-4962	0.15	23	23	0.12	100
CAAALY00014912:2255-2348	0.63	94	26	0.002	81.9
CAAALY00015773:810-859	0.33	50	30	8.10E-06	90
CAAALY00015773:867-967	0.68	101	45	9.10E-15	86.1
CAAALY00016045:2734-2882	1.00	149	41	2.20E-12	81.9
CAAALY00016045:2910-3015	0.71	106	50	9.40E-18	86.8
CAAALY00016045:2910-3015	0.71	106	46	2.30E-15	85.8
CAAALY00018915:287-346	0.40	60	40	8.70E-12	91.7
CAAALY00018915:405-437	0.22	33	29	3.20E-05	97
CAAALY00046511:196-217	0.15	22	22	0.48	100
CAAALY00046511:219-276	0.39	58	26	0.002	86.2
CAAALY00046511:281-330	0.33	50	34	3.30E-08	92
CAAALY00047638:763-921	1.06	159	39	3.50E-11	81.1
CAAALY00047638:763-921	1.06	159	43	1.40E-13	81.8
CAAALY00050863:692-738	0.31	47	23	0.12	87.2
CAAALY00051808:496-570	0.50	75	55	9.80E-21	93.3
CAAALY00066666:49-74	0.17	26	22	0.48	96.2

Supplementary Table S6. Content of repetitive sequences in the chromosome regions of *Xenopus laevis* mapped by *Protopolystoma xenopodis* reads and detected by BLAST using the oocyte-specific 5S rDNA NTS of *X. laevis*.

X. laevis **chromosome:region**	**Number of mapped reads**	**5S rDNA repeat unit position (bp)**	**Copy number of SSRs repeats[1]**	**SSR repeat unit[1]**
6L:164135590-164155982	180,783			
		164136198-164136862	11.2	(AGTTTTCAA)
		164136863-164137564	21.3	(TTTTCAAAG)
			6.3	(TTTTCAACG)
		164137565-164138268	21.3	(TTTTCAAAG)
			6.3	(TTTTCAACG)
		164138269-164138967	21.3	(TTTTCAAAG)
			6.3	(TTTTCAACG)
		164138968-164139687	23.3	(TTTTCAAAG)
		164139688-164140357	6.3	(TTTTCAACG)
		164140358-164141032	16.3	(TTTTCAAAG)
			6.3	(TTTTCAACG)
		164141033-164141714	6.3	(TTTTCAACG)
			6.9	(AGTTTTCAAAGTTTG)
		164141715-164142421	6.3	(TTTTCAACG)
			5.9	(AGTTTTCAAAGTTTG)
		164142422-164143140	22.8	(AAAGTTTTC)
			6.3	(TTTTCAACG)
		164143141-164143859	23.3	(TTTTCAAAG)
			6.3	(TTTTCAACG)
		164143860-164144593	23.3	(TTTTCAAAG)
			6.3	(TTTTCAACG)
		164144594-164145312	25.3	(TTTTCAAAG)
			6.3	(TTTTCAACG)
		164145313-164146031	23.3	(TTTTCAAAG)
			6.3	(TTTTCAACG)
		164146032-164146750	23.3	(TTTTCAAAG)
			6.3	(TTTTCAACG)
		164146751-164147484	23.3	(TTTTCAAAG)
			6.3	(TTTTCAACG)
		164147485-164148554	25.3	(TTTTCAAAG)
			6.3	(TTTTCAACG)
		164148555-164149221	20.3	(TTTTCAAAG)
		164149222-164149968	20.6	(AGTTTTCAA)

			6.3	(TTTTCAACG)
		164149969-164150702	27.3	(TTTTCAAAG)
			6.3	(TTTTCAACG)
		164150703-164151421	25.3	(TTTTCAAAG)
			6.3	(TTTTCAACG)
		164151422-164152140	23.3	(TTTTCAAAG)
			6.3	(TTTTCAACG)
		164152141-164152874	23.3	(TTTTCAAAG)
			6.3	(TTTTCAACG)
		164152875-164153601	25.3	(TTTTCAAAG)
			6.3	(TTTTCAACG)
		164153602-164154320	24.3	(TTTTCAAAG)
			6.3	(TTTTCAACG)
		164154321-164155038	23;3	(TTTTCAAAG)
			6.3	(TTTTCAACG)
		164155039-164155757	23.3	(TTTTCAAAG)
			6.3	(TTTTCAACG)
9_10L:23705318-23743987	218,265			
		23705371-23705873	6.4	(AAGTTTTCAAGGTTGATTTTTTCA)
		23705874-23706571	8.6	(AGTTTTCAAAGTTTCATTTTTCAA)
		23706572-23707269	8.6	(AGTTTTCAAAGTTTCATTTTTCAA)
		23707270-23707772	4.4	(TTTTCAAAGTTTCAT)
		23707773-23708437	10.6	(AAGTTTTCAAAGTTTCATTTTTTA)
		23708438-23709110	8.6	(AGTTTTCAAAGTTTCATTTTTCAA)
		23709111-23709911	13.6	(AGTTTTCAAAGTTTGATTTTTCAA)
		23709912-23710707	11.5	(TTTTCAAAGTTTGAGTTTTCAAAG)
		23710708-23711411	31.3	(TTTTCAAAG)
		23711412-23712071	27.3	(TTTTCAAAG)
		23712072-23712773	21.3	(TTTTCAAAG)
			6.3	(TTTTCAACG)
		23712774-23713468	20.3	(TTTTCAAAG)
			6.3	(TTTTCAACG)
		23713469-23714148	30	(TTTTCAAAG)
		23714149-23714838	9.5	(TTTTCAAAGTTTGAGTTTTCAAAG)
		23714839-23715531	19.3	(TTTTCAAAG)
			6.3	(TTTTCAACG)
		23715532-23716216	9.5	(TTTTCAAAGTTTGATTTTTTAAAG)
		23716217-23716904	9.5	(TTTTCAAAGTTTGAGTTTTCAAAG)

	23716905-23717598	19.3	(TTTTCAAAG)
		6.3	(TTTTCAACG)
	23717599-23719191	56.3	(TTTTCAAAG)
	23719192-23719909	31.3	(TTTTCAAAG)
	23719910-23720613	23.3	(TTTTCAAAG)
		6.3	(TTTTCAACG)
	23720614-23721323	21.3	(TTTTCAAAG)
		6.3	(TTTTCAACG)
	23721324-23722040	22.3	(TTTTCAAAG)
		6.3	(TTTTCAACG)
	23722041-23722743	23.3	(TTTTCAAAG)
		6.3	(TTTTCAACG)
	23722744-23723440	21.3	(TTTTCAAAG)
		6.3	(TTTTCAACG)
	23723441-23724158	20.3	(TTTTCAAAG)
		6.3	(TTTTCAACG)
	23724159-23724862	23.3	(TTTTCAAAG)
		6.3	(TTTTCAACG)
	23724863-23725609	10.5	(TTTTCAAAGTTTGAGTTTTCAAAG)
	23725610-23726280	6.5	(TTTTCAAAGTTTGAGTTTTCAAAG)
		6.3	(TTTTCAACG)
	23726281-23726984	9.5	(TTTTCAAAGTTTGAGTTTTCAAAG)
	23726985-23727688	10.5	(TTTTCAAAGTTTGAGTTTTCAAAG)
	23727689-23728368	10.5	(TTTTCAAAGTTTGAGTTTTCAAAG)
	23728369-23728690	7.9	(AGTTTTCAAAGTTTG)
		6.3	(TTTTCAACG)
	23728691-23729394	13.4	(GTTTTCAAA)
	23729395-23730099	10.5	(TTTTCAAAGTTTGAGTTTTCAAAG)
	23730100-23730803	10.5	(TTTTCAAAGTTTGATTTTTCAAAG)
	23730804-23731513	10.5	(TTTTCAAAGTTTGAGTTTTCAAAG)
	23731514-23732208	10.5	(TTTTCAAAGTTTGAGTTTTCAAAG)
	23732209-23732927	20.3	(TTTTCAAAG)
		6.3	(TTTTCAACG)
	23732928-23733631	23.3	(TTTTCAAAG)
		6.3	(TTTTCAACG)
	23733632-23734317	10.5	(TTTTCAAAGTTTGAGTTTTCAAAG)
	23734318-23735012	20.3	(TTTTCAAAG)
		6.3	(TTTTCAACG)

	23735013-23735731	20.3	(TTTTCAAAG)
		6.3	(TTTTCAACG)
	23735732-23736476	23.3	(TTTTCAAAG)
		6.3	(TTTTCAACG)
	23736477-23737180	9.9	(AGTTTTCAAAGTTTG)
		6.3	(TTTTCAACG)
	23737181-23737899	10.5	(TTTTCAAAGTTTGAGTTTTCAAAG)
	23737900-23738601	23.3	(TTTTCAAAG)
		6.3	(TTTTCAACG)
	23738602-23739313	21.3	(TTTTCAAAG)
		6.3	(TTTTCAACG)
	23739314-23740002	22.3	(TTTTCAAAG)
		6.3	(TTTTCAACG)
	23740003-23740719	19.3	(TTTTCAAAG)
		6.3	(TTTTCAACG)
	23740720-23741422	14.6	(AGTTTTCAA)
		6.3	(TTTTCAACG)
	23741423-23742125	10.5	(TTTTCAAAGTTTGAGTTTTCAAAG)
	23742126-23742814	10.5	(TTTTCAAAGTTTGAGTTTTCAAAG)
	23742815-23743509	19.3	(TTTTCAAAG)
		6.3	(TTTTCAACG)
	23743510-23744205	20.3	(TTTTCAAAG)
		6.3	(TTTTCAACG)

[illegible] Copy number calculation and SSR consensus sequence were obtained by submitting each 5S rDNA repeat unit to an analysis o[illegible] Tandem Repeat Finder software.

Supplementary Table S7. Summary of BLAST searches using short repeats found in the NTS of the oocyte-specific 5S rDNA of *Xenopus laevis* as queries against the genome assembly of *Protopolystoma xenopodis*. BLAST search was configured for short sequences as follows: (a) word size = 7; expected threshold = 1000; match/ mismatch score = 1, -3; gap existence = 5; extension = 2.

Query repeat unit sequence	Number of scaffolds/contigs	Scaffolds/Contigs with more than one repeat	Alignment sizes (bp)	Identity percentage distribution
AGTTTTCAAAGTTTG	4312	98	11 – 15	93.33 – 100
AAGTTTTCAAGGTTGATTTTTTCA	2595	31	11 – 24	87.50 – 100
TTTTCAAAGTTTGAGTTTTCAAAG	2418	73	11 – 24	87.50 – 100

Supplementary Table S8. BLAST searches for the type I 5S rDNA of *Pseudis tocantins* (KX170899) in the genome assemblies of *Bursaphelenchus xylophilus* (assembly accessions numbers: GCA_000231135.1 and GCA_904067135.1).

Genome	Genomic Location (bp)	Orientation	Query start (bp)	Query end (bp)	Alignment Length (bp)	Score	E-val	Identity (%)
GCA_000231135.1	contig05224:139-209	Reverse	1	71	71	39	9.50E-14	88.7
	contig05655:51-121	Reverse	1	71	71	39	9.50E-14	88.7
	contig05870:1104-1174	Forward	1	71	71	39	9.50E-14	88.7
	contig05870:65-135	Reverse	1	71	71	39	9.50E-14	88.7
	contig10893:148-218	Forward	1	71	71	39	9.50E-14	88.7
	contig15042:612-669	Reverse	1	58	58	30	2.20E-08	87.9
	contig17976:164-234	Forward	1	71	71	39	9.50E-14	88.7
	contig18432:90-160	Reverse	1	71	71	39	9.50E-14	88.7
	contig19079:7-68	Reverse	1	62	62	38	3.80E-13	90.3
	contig19459:564-634	Forward	1	71	71	39	9.50E-14	88.7
	contig20618:608-678	Reverse	1	71	71	39	9.50E-14	88.7
	contig20618:77-147	Reverse	1	71	71	39	9.50E-14	88.7
	scaffold00466:205995-206065	Forward	1	71	71	39	9.50E-14	88.7
	scaffold00466:207354-207424	Reverse	1	71	71	39	9.50E-14	88.7
	scaffold00579:737073-737143	Forward	1	71	71	39	9.50E-14	88.7
	scaffold00600:13565-13635	Reverse	1	71	71	39	9.50E-14	88.7
	scaffold00600:19829-19899	Reverse	1	71	71	39	9.50E-14	88.7
	scaffold00600:200285-200355	Reverse	1	71	71	39	9.50E-14	88.7
	scaffold00600:29150-29220	Reverse	1	71	71	39	9.50E-14	88.7
	scaffold00600:39352-39422	Forward	1	71	71	39	9.50E-14	88.7
	scaffold00600:42156-42226	Reverse	1	71	71	39	9.50E-14	88.7
	scaffold00600:488772-488842	Forward	1	71	71	39	9.50E-14	88.7
	scaffold00600:53050-53120	Forward	1	71	71	39	9.50E-14	88.7
	scaffold00600:54433-54503	Reverse	1	71	71	39	9.50E-14	88.7
	scaffold00600:56698-56768	Forward	1	71	71	39	9.50E-14	88.7
	scaffold00600:6769-6839	Forward	1	71	71	39	9.50E-14	88.7
	scaffold00647:73664-73734	Reverse	1	71	71	35	2.30E-11	87.3
	scaffold00647:87541-87611	Reverse	1	71	71	39	9.50E-14	88.7
	scaffold00647:95875-95945	Forward	1	71	71	39	9.50E-14	88.7
	scaffold00865:900-970	Forward	1	71	71	39	9.50E-14	88.7
	scaffold00921:4078-4148	Reverse	1	71	71	39	9.50E-14	88.7
	scaffold01280:3890-3960	Reverse	1	71	71	39	9.50E-14	88.7

	scaffold01280:851-921	Reverse	1	71	71	39	9.50E-14	88.7
	scaffold01304:3621-3691	Reverse	1	71	71	39	9.50E-14	88.7
	scaffold01304:4770-4840	Forward	1	71	71	39	9.50E-14	88.7
	scaffold01445:2478-2548	Forward	1	71	71	39	9.50E-14	88.7
	scaffold01656:114273-114343	Reverse	1	71	71	39	9.50E-14	88.7
	scaffold01656:136177-136247	Reverse	1	71	71	39	9.50E-14	88.7
	scaffold01656:1829-1899	Forward	1	71	71	39	9.50E-14	88.7
	scaffold01656:28853-28923	Reverse	1	71	71	39	9.50E-14	88.7
	scaffold01656:31912-31982	Reverse	1	71	71	39	9.50E-14	88.7
	scaffold01656:34194-34255	Forward	1	62	62	38	3.80E-13	90.3
	scaffold01656:35484-35554	Reverse	1	71	71	39	9.50E-14	88.7
GCA_904067135.1	BXYJ5_Chr1:1174445-1174515	Forward	1	71	71	39	9.50E-14	88.7
	BXYJ5_Chr1:1174976-1175046	Forward	1	71	71	39	9.50E-14	88.7
	BXYJ5_Chr1:1179618-1179688	Reverse	1	71	71	39	9.50E-14	88.7
	BXYJ5_Chr1:1201212-1201282	Forward	1	71	71	39	9.50E-14	88.7
	BXYJ5_Chr1:1201744-1201814	Forward	1	71	71	39	9.50E-14	88.7
	BXYJ5_Chr1:1289179-1289249	Forward	1	71	71	39	9.50E-14	88.7
	BXYJ5_Chr1:1290478-1290539	Reverse	1	62	62	38	3.80E-13	90.3
	BXYJ5_Chr1:1292751-1292821	Forward	1	71	71	39	9.50E-14	88.7
	BXYJ5_Chr1:1296233-1296303	Forward	1	71	71	39	9.50E-14	88.7
	BXYJ5_Chr1:1302622-1302692	Forward	1	71	71	39	9.50E-14	88.7
	BXYJ5_Chr1:1329253-1329323	Reverse	1	71	71	39	9.50E-14	88.7
	BXYJ5_Chr1:1331987-1332057	Reverse	1	71	71	39	9.50E-14	88.7
	BXYJ5_Chr1:1334534-1334604	Reverse	1	71	71	39	9.50E-14	88.7
	BXYJ5_Chr1:1335066-1335136	Reverse	1	71	71	39	9.50E-14	88.7
	BXYJ5_Chr1:1335597-1335667	Reverse	1	71	71	39	9.50E-14	88.7
	BXYJ5_Chr1:1336128-1336198	Reverse	1	71	71	39	9.50E-14	88.7
	BXYJ5_Chr1:1336659-1336729	Reverse	1	71	71	39	9.50E-14	88.7
	BXYJ5_Chr1:1337190-1337260	Reverse	1	71	71	39	9.50E-14	88.7
	BXYJ5_Chr1:1337721-1337791	Reverse	1	71	71	39	9.50E-14	88.7
	BXYJ5_Chr1:1338252-1338322	Reverse	1	71	71	39	9.50E-14	88.7
	BXYJ5_Chr1:1338783-1338853	Reverse	1	71	71	39	9.50E-14	88.7
	BXYJ5_Chr1:1339314-1339384	Reverse	1	71	71	39	9.50E-14	88.7
	BXYJ5_Chr1:1339845-1339915	Reverse	1	71	71	39	9.50E-14	88.7
	BXYJ5_Chr1:1340376-1340446	Reverse	1	71	71	39	9.50E-14	88.7
	BXYJ5_Chr1:1340907-1340977	Reverse	1	71	71	39	9.50E-14	88.7

BXYJ5_Chr1:1341438-1341508	Reverse	1	71	71	39	9.50E-14	88.7
BXYJ5_Chr1:1341969-1342039	Reverse	1	71	71	39	9.50E-14	88.7
BXYJ5_Chr1:1342500-1342570	Reverse	1	71	71	39	9.50E-14	88.7
BXYJ5_Chr1:1343031-1343101	Reverse	1	71	71	39	9.50E-14	88.7
BXYJ5_Chr1:1343562-1343632	Reverse	1	71	71	39	9.50E-14	88.7
BXYJ5_Chr1:1344093-1344163	Reverse	1	71	71	39	9.50E-14	88.7
BXYJ5_Chr1:1344624-1344694	Reverse	1	71	71	39	9.50E-14	88.7
BXYJ5_Chr1:1345155-1345225	Reverse	1	71	71	39	9.50E-14	88.7
BXYJ5_Chr1:1345686-1345756	Reverse	1	71	71	39	9.50E-14	88.7
BXYJ5_Chr1:1346217-1346287	Reverse	1	71	71	39	9.50E-14	88.7
BXYJ5_Chr1:1346748-1346818	Reverse	1	71	71	39	9.50E-14	88.7
BXYJ5_Chr1:1347279-1347349	Reverse	1	71	71	39	9.50E-14	88.7
BXYJ5_Chr1:1347810-1347880	Reverse	1	71	71	39	9.50E-14	88.7
BXYJ5_Chr1:1348341-1348411	Reverse	1	71	71	39	9.50E-14	88.7
BXYJ5_Chr1:1348872-1348942	Reverse	1	71	71	39	9.50E-14	88.7
BXYJ5_Chr1:1349403-1349473	Reverse	1	71	71	39	9.50E-14	88.7
BXYJ5_Chr1:1349934-1350004	Reverse	1	71	71	39	9.50E-14	88.7
BXYJ5_Chr1:1350465-1350535	Reverse	1	71	71	39	9.50E-14	88.7
BXYJ5_Chr1:1350996-1351066	Reverse	1	71	71	39	9.50E-14	88.7
BXYJ5_Chr1:1351527-1351597	Reverse	1	71	71	39	9.50E-14	88.7
BXYJ5_Chr1:1352058-1352128	Reverse	1	71	71	39	9.50E-14	88.7
BXYJ5_Chr1:1352589-1352659	Reverse	1	71	71	39	9.50E-14	88.7
BXYJ5_Chr1:1353120-1353190	Reverse	1	71	71	39	9.50E-14	88.7
BXYJ5_Chr1:1353651-1353721	Reverse	1	71	71	39	9.50E-14	88.7
BXYJ5_Chr1:1354182-1354252	Reverse	1	71	71	39	9.50E-14	88.7
BXYJ5_Chr1:1354713-1354783	Reverse	1	71	71	39	9.50E-14	88.7
BXYJ5_Chr1:1355244-1355314	Reverse	1	71	71	39	9.50E-14	88.7
BXYJ5_Chr1:1355775-1355845	Reverse	1	71	71	39	9.50E-14	88.7
BXYJ5_Chr1:1356306-1356376	Reverse	1	71	71	39	9.50E-14	88.7
BXYJ5_Chr1:1356837-1356907	Reverse	1	71	71	39	9.50E-14	88.7
BXYJ5_Chr1:1357368-1357438	Reverse	1	71	71	39	9.50E-14	88.7
BXYJ5_Chr1:1357899-1357969	Reverse	1	71	71	39	9.50E-14	88.7
BXYJ5_Chr1:1358430-1358500	Reverse	1	71	71	39	9.50E-14	88.7
BXYJ5_Chr1:1358961-1359031	Reverse	1	71	71	39	9.50E-14	88.7
BXYJ5_Chr1:1359492-1359562	Reverse	1	71	71	39	9.50E-14	88.7
BXYJ5_Chr1:1360023-1360093	Reverse	1	71	71	39	9.50E-14	88.7

BXYJ5_Chr1:1360554-1360624	Reverse	1	71	71	39	9.50E-14	88.7
BXYJ5_Chr1:1361085-1361155	Reverse	1	71	71	39	9.50E-14	88.7
BXYJ5_Chr1:1361616-1361686	Reverse	1	71	71	39	9.50E-14	88.7
BXYJ5_Chr1:1362147-1362217	Reverse	1	71	71	39	9.50E-14	88.7
BXYJ5_Chr1:1367929-1367999	Forward	1	71	71	39	9.50E-14	88.7
BXYJ5_Chr1:1377292-1377362	Reverse	1	71	71	39	9.50E-14	88.7
BXYJ5_Chr1:1378441-1378511	Forward	1	71	71	39	9.50E-14	88.7
BXYJ5_Chr1:1386750-1386820	Forward	1	71	71	39	9.50E-14	88.7
BXYJ5_Chr1:1395191-1395261	Reverse	1	71	71	39	9.50E-14	88.7
BXYJ5_Chr1:1401458-1401528	Reverse	1	71	71	39	9.50E-14	88.7
BXYJ5_Chr1:1413411-1413481	Reverse	1	71	71	39	9.50E-14	88.7
BXYJ5_Chr1:1427802-1427872	Forward	1	71	71	39	9.50E-14	88.7
BXYJ5_Chr1:1432908-1432978	Reverse	1	71	71	39	9.50E-14	88.7
BXYJ5_Chr1:1434108-1434178	Forward	1	71	71	39	9.50E-14	88.7
BXYJ5_Chr1:1443734-1443804	Forward	1	71	71	39	9.50E-14	88.7
BXYJ5_Chr1:1444885-1444955	Reverse	1	71	71	39	9.50E-14	88.7
BXYJ5_Chr1:1445924-1445994	Forward	1	71	71	39	9.50E-14	88.7
BXYJ5_Chr1:1447306-1447376	Reverse	1	71	71	39	9.50E-14	88.7
BXYJ5_Chr1:1449571-1449641	Forward	1	71	71	39	9.50E-14	88.7
BXYJ5_Chr1:1606403-1606473	Reverse	1	71	71	39	9.50E-14	88.7
BXYJ5_Chr1:1965015-1965085	Forward	1	71	71	39	9.50E-14	88.7
BXYJ5_Chr3:2743438-2743508	Forward	1	71	71	39	9.50E-14	88.7
BXYJ5_Chr3:2744797-2744867	Reverse	1	71	71	39	9.50E-14	88.7
BXYJ5_Chr3:618068-618138	Reverse	1	71	71	35	2.30E-11	87.3
BXYJ5_Chr3:635074-635144	Reverse	1	71	71	39	9.50E-14	88.7
BXYJ5_Chr3:643365-643435	Forward	1	71	71	39	9.50E-14	88.7
BXYJ5_Chr6:4855260-4855330	Forward	1	71	71	39	9.50E-14	88.7
BXYJ5_Chr6:5043999-5044069	Forward	1	71	71	39	9.50E-14	88.7
BXYJ5_Chr6:5045936-5046006	Forward	1	71	71	39	9.50E-14	88.7
BXYJ5_Chr6:5047873-5047943	Forward	1	71	71	39	9.50E-14	88.7
BXYJ5_Chr6:5049810-5049880	Forward	1	71	71	39	9.50E-14	88.7
BXYJ5_Chr6:5051747-5051817	Forward	1	71	71	39	9.50E-14	88.7
BXYJ5_Chr6:5052658-5052728	Forward	1	71	71	39	9.50E-14	88.7
BXYJ5_Chr6:5053569-5053639	Forward	1	71	71	39	9.50E-14	88.7
BXYJ5_Chr6:5055506-5055576	Forward	1	71	71	39	9.50E-14	88.7
BXYJ5_Chr6:5057443-5057513	Forward	1	71	71	39	9.50E-14	88.7

	BXYJ5_Chr6:5058354-5058424	Forward	1	71	71	39	9.50E-14	88.7
	BXYJ5_Chr6:5059264-5059334	Forward	1	71	71	39	9.50E-14	88.7
	BXYJ5_Chr6:5060175-5060245	Forward	1	71	71	39	9.50E-14	88.7
	BXYJ5_Chr6:5061086-5061156	Forward	1	71	71	39	9.50E-14	88.7
	BXYJ5_Chr6:5061996-5062066	Forward	1	71	71	39	9.50E-14	88.7
	BXYJ5_Chr6:5062907-5062977	Forward	1	71	71	39	9.50E-14	88.7
	BXYJ5_Chr6:5063817-5063887	Forward	1	71	71	39	9.50E-14	88.7
	BXYJ5_Chr6:5064728-5064798	Forward	1	71	71	39	9.50E-14	88.7
	BXYJ5_Chr6:5065639-5065709	Forward	1	71	71	39	9.50E-14	88.7
	BXYJ5_Chr6:5066550-5066620	Forward	1	71	71	39	9.50E-14	88.7
	BXYJ5_Chr6:5067461-5067531	Forward	1	71	71	39	9.50E-14	88.7
	BXYJ5_Chr6:5068371-5068441	Forward	1	71	71	39	9.50E-14	88.7
	BXYJ5_Chr6:5069281-5069351	Forward	1	71	71	39	9.50E-14	88.7
	BXYJ5_Chr6:5070191-5070261	Forward	1	71	71	39	9.50E-14	88.7
